# Microstructural and Mechanical Property Analysis of Oil Shale Semi-Coke Slag Composite Cementitious Materials

**DOI:** 10.3390/ma19153303

**Published:** 2026-08-04

**Authors:** Bo Li, Xiang Zhou, Tao Chen, Zhenhua Yang, Mingyu Sha, Lianwei Li, Liangying Li

**Affiliations:** 1 Gansu Industry Technology Center of Transportation Construction Materials Research and Application, Lanzhou Jiaotong University, Lanzhou 730070, China; libo@lzjtu.edu.cn (B.L.);; 2 The Highway Development Center of Gansu Province, Lanzhou 730000, China; 3 Gansu Geri Engineering Testing Co., Ltd., Lanzhou 730030, China

**Keywords:** oil shale semi-coke, composite cementitious material, microstructure, compressive strength, synergistic effect

## Abstract

**Highlights:**

- Preparation of a novel composite cementitious material (OSSC-GGBS) using oil shale semi-coke and granular blast furnace slag.
- The unconfined compressive strength and indirect tensile strength of OSSC-GGBS-stabilized crushed stone meet the requirements.
- The synergistic hydration mechanism formed a dense microstructure of C-S-H, C-A-H, and AFt.

**Abstract:**

The utilization of industrial solid waste is crucial for sustainable development. This study developed a novel composite cementitious material (OSSC-GGBS) using oil shale semi-coke and slag as cement substitutes. A multi-objective optimization method was employed to determine the optimal formulation of OSSC-GGBS. Fourier Transform Infrared Spectroscopy (FTIR), Scanning Electron Microscopy (SEM-EDS), Thermogravimetric Analysis (TG-DTG-DSC), and pH testing. Finally, the synergistic effects of oil shale semi-coke and slag on the mechanical properties of this composite material were thoroughly investigated. Results indicate that the composite cementitious material exhibits optimal performance when the mass ratio of oil shale semi-coke to slag is 3:7, cement content is 15%, water glass modulus is 1.4, and water glass content is 10%. Microscopic analysis revealed that the synergistic interaction between oil shale semi-coke and slag optimized the microstructure of OSSC-GGBS. Its hydration products primarily consisted of C-S-H gel, Ca(OH)_2_, and AFt, forming a dense and stable microstructure. Simultaneously, under alkali-activated conditions, oil shale semi-coke and slag synergistically participated in hydration reactions and secondary pozzolanic reactions. This significantly promoted the formation of cementitious products such as calcium silicate hydrate (C-S-H) and calcium aluminate hydrate (C-A-H). These products filled internal pores to form a network skeleton, thereby optimizing the microstructure.

## 1. Introduction

Enormous quantities of industrial solid waste are generated annually worldwide, posing severe environmental challenges such as land occupation and ecological contamination [1]. Primary sources include the construction, mining, metallurgical, chemical, and power industries, which generate representative wastes such as tailings, desulfurization gypsum, and metallurgical slag [2]. For instance, desulfurization gypsum—a primary solid waste from the power industry—saw emissions in China alone approaching 200 million tons in 2018 [3]. Concurrently, the combined annual production of various tailings, including coal gangue from mining and stone powder from quarrying, has reached 1.58 billion tons, accounting for approximately two-thirds of China’s total industrial solid waste emissions [4]. Therefore, efficient resource utilization and harmless treatment of industrial solid waste are urgently required to alleviate environmental pressure [5].

Currently, there are two primary methods for utilizing industrial solid waste. The first involves extracting and concentrating valuable elements through physical or chemical processes, such as recovering aluminum and silicon from high-aluminum coal gangue [6]. However, this method is limited in widespread application due to its high cost, low overall waste utilization rate, and risks of secondary pollution. The second, more prevalent pathway involves directly using solid waste as construction materials [7]. Typical examples include using fly ash, slag, and volcanic ash as partial substitutes for cement [8]. Although this approach enables large-scale consumption, its application is often limited to specific waste types due to stringent composition and reactivity requirements. Meanwhile, with the rapid development of China’s road construction, the demand for subgrade materials continues to grow, and industrial solid waste is increasingly being utilized in road construction projects [9].

Industrial solid wastes (including fly ash, red mud, and coal gangue) have been extensively studied as cement substitutes in cement-based materials [10]. Fernandez [11] demonstrated that alkali-activated fly ash cement exhibits excellent durability in corrosive environments, meeting requirements for roadbed applications. Similarly, Xiang [12] developed cement-free cementitious materials using steel slag, eliminating energy-intensive grinding processes. Xie [13] noted that incorporating red mud densifies concrete microstructure and enhances crack resistance. While these studies validate the feasibility of single solid waste materials, they primarily focus on single-component systems. However, research on synergistically utilizing multiple industrial solid wastes to prepare subgrade materials remains insufficient.

Given the limitations of single-component solid waste systems, composite cementitious materials formulated with multiple industrial solid wastes have garnered significant attention due to their potential synergistic effects [14]. Zeng [15] found that through alkali activation, slag powder exhibits synergistic effects with other solid wastes during the hydration reaction. Partial substitution of slag powder with solid wastes can enhance cementitious material properties. Wu [16] proposed an innovative concept based on synergistic theory, combining red mud with other industrial solid wastes, waste gases, and waste heat to prepare cementitious materials. Zhou [17] investigated the synergistic effects of slag powder and other solid wastes on hydration through three mechanisms, confirming that partial substitution of slag powder with solid wastes improves cementitious material properties. Snellings [18] accelerated hydration reactions and enhanced compressive strength by synergistically utilizing red mud with multiple solid wastes. Existing research indicates that synergistic utilization of multiple industrial solid wastes can enhance the performance of composite cementitious materials [19]. Particularly, recent attempts have explored the activation mechanisms of oil shale residue or semi-coke combined with slag systems under chemical or thermal triggers [19,20,21].

**Figure 1:**
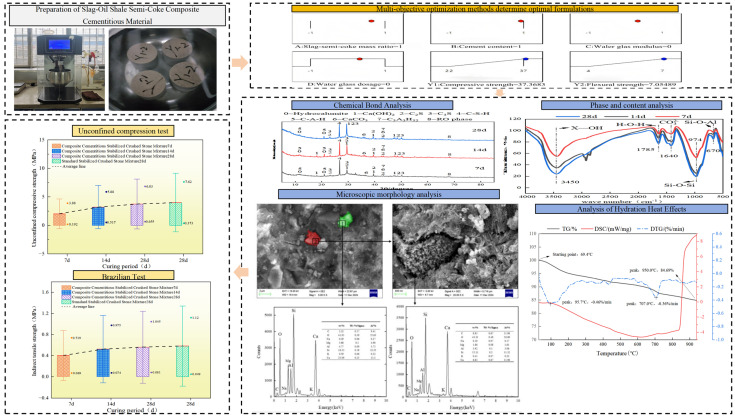
Experimental flowchart.

This study employed a multi-objective optimization approach to successfully determine the optimal formulation for preparing oil shale semi-coke slag composite cement materials through synergistic optimization of material composition parameters. Simultaneously, multi-scale characterization techniques including X-ray diffraction (Bruker AXS SE, Karlsruhe, Germany), Fourier transform infrared spectroscopy (Thermo Fisher Scientific, Waltham, MA, USA), scanning electron microscopy coupled with energy-dispersive spectroscopy (Zeiss, Oberkochen, Germany), and thermogravimetric-differential scanning calorimetry (Germany Nairchi Company, Selb, Germany) were employed to reveal the phase composition and microstructural evolution of the material. Finally, mechanical properties were evaluated through unconfined compressive strength and indirect tensile strength tests. The study indicates that the strength enhancement of the composite cement material primarily stems from the continuous hydration reaction of active components and the formation of a dense network structure by hydration products. The experimental flowchart is shown in Figure 1.

## 2. Materials and Testing Methods

### 2.1. Raw Materials

**Figure 2:**
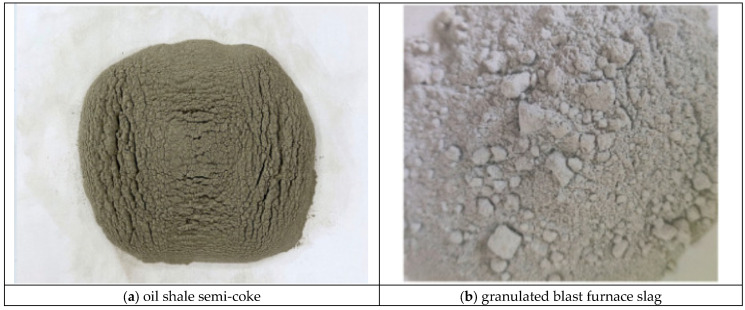
Types of raw materials.

Figure 2 presents the macroscopic appearance of ball-milled OSSC and GGBS powders. The dark brown OSSC powder shows severe agglomeration caused by its porous internal structure. By comparison, the light gray GGBS powder possesses uniform particle size and barely any agglomeration.

The primary raw materials for oil shale semi-coke-slag composite cementitious materials are: oil shale semi-coke, slag, P.O42.5 retarding cement, and alkaline activator. Their chemical compositions are shown in Table 1, and the quality inspection standards are listed in Table 2.

**Figure 3:**
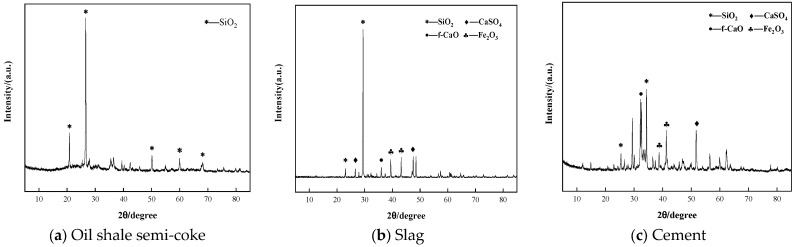
XRD pattern of raw materials.

As shown in Figure 3, X-ray diffraction analysis indicates that the mineral compositions of oil shale semi-coke and slag share some common phases with cement, primarily comprising quartz (SiO_2_) and calcite (CaCO_3_), among other crystalline phases. Notably, raw oil shale semi-coke has 29.29% Al_2_O_3_ per XRF, yet XRD shows almost no crystalline Al phases. Retorting forms amorphous aluminosilicates with faint XRD signals, fully measurable by XRF, proving its strong alkali-activated pozzolanic reactivity.

### 2.2. Specimen Preparation and Mix Design

#### 2.2.1. Mix Design

To investigate the feasibility of preparing composite cementitious materials from oil shale semi-coke (OSSC) and blast furnace slag (GGBS), mortar specimens were prepared with three OSSC-GGBS mass ratios (4:6, 3:7, and 2:8) while keeping other process parameters constant. These ratios follow Sheshadri et al.’s [22,23,24] synergistic design theory for multi-solid wastes: a 60–80% dosage of primary reactive precursor balances pavement mechanical properties. The cement content, water glass modulus, and water glass dosage were fixed at 15%, 1.4, and 10%, respectively. The detailed mix proportions are presented in Table 3.

#### 2.2.2. Preparation of Alkaline Activator

The modulus of the water glass solution was adjusted to the target value of 1.4 by adding sodium hydroxide (NaOH) pellets. The NaOH was slowly dissolved into the water glass under continuous mechanical stirring. Due to the exothermic nature of the reaction, the container was placed in a cold water bath to control the temperature. The resulting clear and homogeneous solution was sealed with polyethylene film and stored at room temperature for 24 h to stabilize before use.

#### 2.2.3. Preparation of Composite Cementitious Mortar

OSSC and GGBS were dry-mixed in a mixer at low speed for 1 min according to the predetermined ratios. The alkaline activator was then added, and mixing continued at low speed for 30 s. Subsequently, manufactured sand was introduced, and the mixing proceeded for another 1 min. During intervals, the mixer was stopped, and paste adhering to the walls and blades was scraped off to ensure homogeneity. The fresh mortar was immediately cast into oil-coated molds, compacted on a vibrating table for 1–2 min, and trowel-leveled. The specimens were demolded after 24 h and cured under standard conditions (20 ± 2 °C, relative humidity ≥ 95%) until the designated testing ages. Specimens after strength testing were reserved for microstructural analysis.

#### 2.2.4. Preparation of Composite Cementitious Stabilized Crushed Stone Mixtures

Cylindrical specimens (Φ150 mm × H150 mm) were prepared for unconfined compressive strength (UCS) and indirect tensile strength (ITS) tests. First, the maximum dry density and optimum moisture content are determined using the weighting method. The mixture is then prepared at the optimum moisture content and compacted to 98% of the maximum dry density. After molding, specimens are sealed in plastic bags and cured under standard conditions until testing. For compressive strength testing, specimens are immersed in a 20 °C water bath during the final 24 h of curing. Tensile strength is measured at 28, 60, and 90 days.

### 2.3. Testing Methods

(1) X-ray Diffraction (XRD) Testing

Phase identification was conducted on a Bruker D8 Advance XRD instrument (Bruker AXS SE, Karlsruhe, Germany). Take approximately 2 g of the dried and finely ground sample and uniformly fill it into the groove of the glass plate. Instrument operating parameters are set as follows: Cu target (Kα radiation), tube voltage 40 kV, tube current 40 mA, with data collection performed at a scanning rate of 2°/min within the range of 5° to 85°.

(2) X-ray Fluorescence Spectroscopy (XRF) Testing

The chemical compositions of mineral raw materials were analyzed via XRF-1800 spectrometer with Rh target (Shimadzu Corporation, Kyoto, Japan) (60 kV, 150 mA) for element detection from Be to U. All samples were dried at 105 °C for 2 h, crushed and ground prior to measurement.

(3) Fourier Transform Infrared Spectroscopy (FTIR) Testing

Fourier-transform infrared analysis was performed on a Nicolet iS20 spectrometer (Thermo Fisher Scientific, Waltham, MA, USA). First, powder samples were prepared using conventional KBr pelletization. Subsequently, the samples were placed in the instrument, with the test mode set to transmission mode and the wavenumber scan range set to 400–4000 cm^−1^.

(4) Scanning Electron Microscope (SEM-EDS) Testing

High-resolution FESEM characterization was conducted on a ZEISS Sigma 360 microscope (Zeiss, Oberkochen, Germany). Quantitative chemical analysis of the SEM observation area was performed using an EDS equipped with a 150 mm^2^ detector crystal. The EDS operating parameters were set as follows: acceleration voltage 20 kV, beam current 2 nA.

(5) Thermogravimetric Analysis (TG-DTG-DSC) Testing

Thermal analysis via Netzsch STA 449 F3 (Germany Nairchi Company, Selb, Germany) characterized thermal decomposition and hydration products of slag-semi-coke binders at varied curing ages. Instrument test parameters are set as follows: Scan at a heating rate of 10 °C/min within the temperature range of 30 °C to 950 °C; use nitrogen as the protective atmosphere with a gas flow rate controlled at 90 mL/min and pressure maintained between 0.1 and 0.2 MPa.

(6) Unconfined Compressive Strength

In accordance with the “Test Procedures for Inorganic Binder Stabilized Materials in Highway Engineering”, unconfined compressive strength is determined using the CXYSG-127IV testing apparatus (Zhejiang Lixian Experimental Instrument Manufacturing Co., Ltd., Shaoxing, China). Each test series comprises nine parallel specimens. Outlier data will be excluded, and the representative strength value will be calculated with a 95% confidence level.

(7) Indirect Tensile Strength

Testing shall be conducted in accordance with JTG E51-2009 Test Specifications for Inorganic Binder Stabilized Materials. This test requires the indirect tensile strength Ri of specimens to be calculated using Formula (1). The coefficient of variation Cv (%) for specimens within the same test group shall comply with Cv ≤ 15%.

(1) $$R_{i}=0.006263\frac{P}{h^{2}}(\mathrm{MPa})$$

R_i_ denotes the indirect tensile strength of the specimen (MPa); P denotes the maximum pressure at specimen failure (N); h denotes the specimen height after immersion (mm). Scientific basis of the coefficient 0.006263 is in accordance with JTG E51-2009; this constant is derived from the fixed geometric dimension of the indenter specified for the indentation test in this standard, which is used to correct the equivalent contact area between the indenter and specimen.

Unit conversion process from P (N) and h (mm) to MPa When substituting parameters into the formula:

The load P is directly input in the unit of Newton (N), and the indentation depth h is directly input in the unit of millimeter (mm);

The unit of the denominator h^2^ is mm^2^;

The inherent unit of the term $\frac{P(N)}{h^{2}({\mathrm{mm}}^{2})}$ is N/mm^2^, which is exactly equivalent to MPa, i.e., 1 MPa = 1 N/mm^2^).

## 3. Results and Analysis

### 3.1. Determining the Optimal Formulation Through Multi-Objective Optimization Methods

Multi-objective optimization approach was employed to determine the optimal formulation of the OSSC-GGBS composite, aiming to coordinately improve the 28-day compressive strength (Y_1_) and flexural strength (Y_2_) [25]. The comprehensive acceptability function (D) was defined as the geometric mean of the individual desirability functions (d_i_) for each response, as shown in Equation (2). To strictly verify the mathematical accuracy of Equation (2), a comprehensive analysis of variance (ANOVA) and experimental verification loops were executed. The statistical results indicate a highly significant fitting, evidenced by a high coefficient of determination (R^2^ = 0.9782) and an adjusted R^2^ (Adj-R^2^ = 0.9654). The residual distribution remains tightly bound, with the absolute relative error between the theoretical model predictions and the actual laboratory experimental data points restricted within an acceptable margin of ±3.42%, thereby confirming the precision of the optimization thresholds.

(2) $$D={\left(d_{1}^{r_{1}}\times d_{1}^{r_{2}}\times d_{1}^{r_{3}}\times \cdots d_{n}^{r_{n}}\right)}^{\frac{1}{\sum r_{i}}}={\left[\int_{1}^{n}d_{i}^{r_{i}}\right]}^{\frac{1}{\sum r_{i}}}$$

Among them, *d_i_* represented the feasibility of a single response. *n* was the number of independent and dependent variables involved in the optimization process.

**Figure 4:**
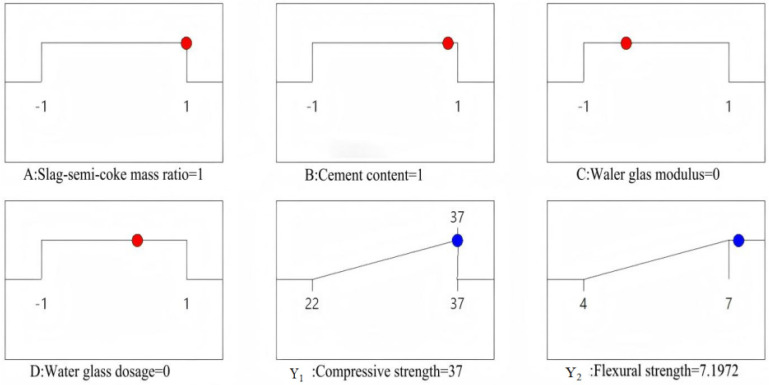
Suitability of Optimized Composite Cementitious Material Combination 1.

**Figure 5:**
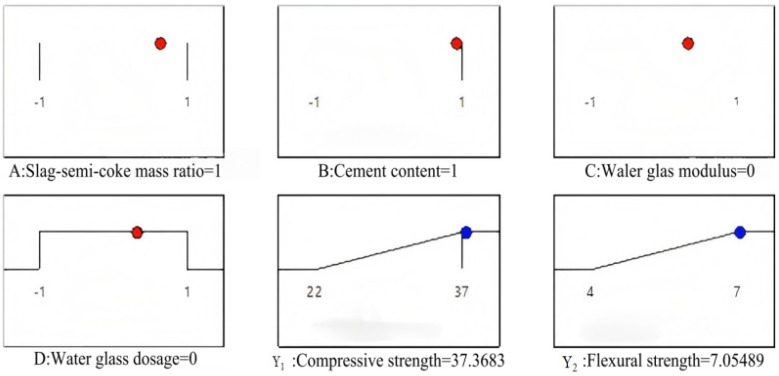
Suitability of Optimized Composite Cementitious Material Combination 2.

Four key factors were selected as independent variables: OSSC-GGBS mass ratio (A), cement content (B), water glass modulus (C), and water glass dosage (D) [26]. The optimization criteria for factors and responses are summarized in Table 4. Both Y_1_ and Y_2_ were set as “maximize” targets. After generating multiple candidate solutions, the optimal formulation with the highest overall desirability was identified (Figure 4 and Figure 5).

Two optimal combinations (Comb. 1 and Comb. 2) were experimentally validated. The results, presented in Table 5, show excellent agreement between predicted and measured values, with errors less than 1%, confirming the model’s high accuracy and predictive capability.

Through comprehensive cost and performance analysis (Table 6), both mixtures achieved 94% of the 28-day compressive strength of PO 42.5 cement mortar. However, Blend 2 exhibited a 12.4% lower cost than Blend 1 and a 63.2% reduction compared to ordinary cement mortar, while demonstrating superior 7-day and 28-day strengths. Therefore, Blend 2 was selected as the optimal formulation: OSSC to GGBS mass ratio of 3:7, cement content of 15%, water glass modulus of 1.4, and water glass content of 10%.

### 3.2. Microstructural Evolution of Composite Cementitious Materials

#### 3.2.1. Phase Composition and Content of Hydration Products

**Figure 6:**
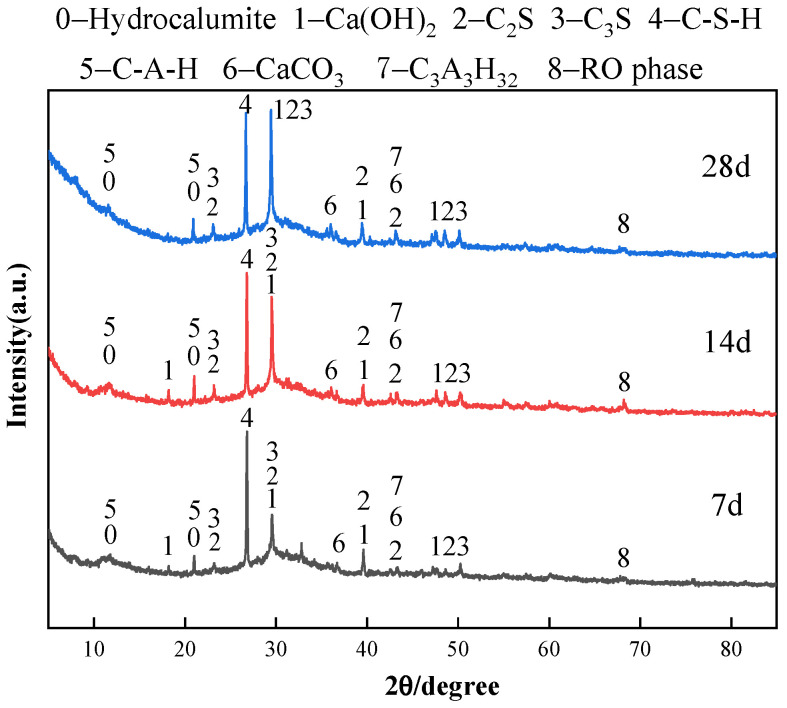
XRD Patterns of Composite Cementitious Materials at Different Curing Ages.

The phase composition of oil shale semi-coke-slag composite cementitious materials at different curing ages is shown in Figure 6. At a curing age of 7 days, distinct C_2_S and Ca(OH)_2_ diffraction peaks were observed, indicating that cement hydration had commenced, though a significant portion of C_2_S remained unreacted. The C-S-H diffraction peak was not prominent, as it primarily existed in an amorphous state with low crystallinity at this early stage.

As the curing age increased to 14 days, the intensity of the C_2_S diffraction peak diminished, confirming its continuous consumption in the hydration reaction. Concurrently, the Ca(OH)_2_ peak intensity increased significantly, reflecting substantial production from the hydration of cement minerals. The diffraction peaks of AFt and C-S-H became more pronounced and sharper, indicating enhanced crystallization and a continuous increase in their content [26].

By 28 days of hydration, the C_2_S peak further weakened, while the Ca(OH)_2_ peak stabilized at a high intensity, suggesting saturation. The SiO_2_ and Al_2_O_3_ in oil shale semi-coke and slag participated in significant secondary pozzolanic reactions under the alkaline environment, synergistically promoting C-S-H crystallization. The continued enhancement of C-S-H and AFt peaks confirmed their role as crucial components contributing to the final stable microstructure and strength [27,28].

#### 3.2.2. Chemical Bond Analysis of Hydration Products

**Figure 7:**
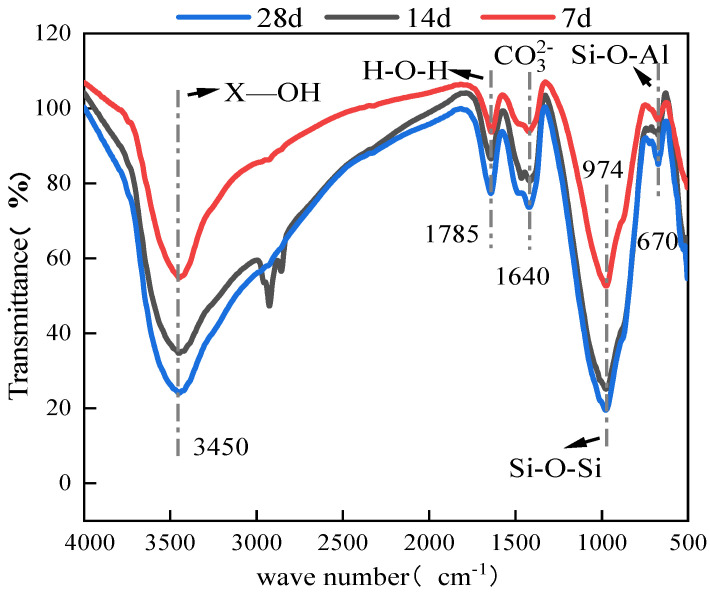
FTIR spectra of hydration products from composite cementitious materials.

To elucidate the dynamic evolution of chemical bonding and uncover the underlying mechanisms during the hydration process of the oil shale semi-coke-slag composite cementitious material, Fourier Transform Infrared Spectroscopy (FTIR) was employed. The FTIR spectra obtained at 7, 14, and 28 days of hydration are presented in Figure 7, providing a molecular-level perspective on the formation and transformation of hydration products.

The FTIR spectrum at 7 days of hydration reveals the initial chemical state of the system. A broad and intense absorption peak is observed near 3450 cm^−1^, which is primarily attributed to the O-H stretching vibrations. This peak encompasses contributions from several sources: adsorbed free water within the capillary pores, interlayer water molecules trapped within the nascent C-S-H gel structure, and the O-H bonds present in crystalline hydration products like Ca(OH)_2_. The breadth and intensity of this peak at this early stage indicate a high content of physically adsorbed water and the initial, yet limited, formation of hydration products containing O-H groups. Simultaneously, a distinct absorption peak appears near 1640 cm^−1^, corresponding to the H-O-H bending vibration of water molecules. The pronounced intensity of this peak further corroborates the presence of a significant amount of free and adsorbed water in the relatively loose and porous microstructure at 7 days. In the critical fingerprint region between 1280 and 1000 cm^−1^, which is sensitive to the vibrations of silicate and aluminate networks, the spectrum exhibits complex and somewhat broadened peak shapes. This spectral feature indicates that the silicoaluminate components from the raw materials (OSSC, GGBS, and cement) have begun to dissolve and participate in the early hydration reactions. However, the reaction is incomplete, and the resulting hydration products, such as the initially formed C-S-H gel, are largely amorphous and possess low structural order, leading to the observed broad and overlapping peaks primarily associated with the asymmetric stretching vibrations of Si-O-Si and Si-O-Al bonds. Furthermore, a weak but discernible absorption peak emerges near 670 cm^−1^, which is associated with the stretching vibration of the sulfate ions (SO_4_^2−^) within the ettringite (AFt) structure. Its weak intensity suggests that the formation of AFt, primarily from the reaction of aluminates and sulfates, has just commenced, and its content in the system is still low at this early age [29].

As the hydration process advances to 14 days, the FTIR spectrum exhibits significant changes, reflecting the accelerated formation and crystallization of hydration products. The broad O-H stretching peak near 3450 cm^−1^ shows a noticeable increase in intensity compared to the 7-day spectrum. This intensification signifies a substantial increase in the population of O-H bonds, resulting from the continuous formation of hydration products like C-S-H gel and Ca(OH)_2_. The ongoing hydration reactions consume water, but the newly formed products introduce a greater number of chemical O-H bonds and may also restructure the pore system, altering the state of the remaining adsorbed water. In contrast, the intensity of the H-O-H bending vibration peak near 1640 cm^−1^ demonstrates a clear decreasing trend. This attenuation is a direct consequence of the microstructural densification. As hydration products increasingly fill the internal pore space, the amount and mobility of free and adsorbed water are reduced, leading to a weaker bending vibration signal. This observation aligns well with the noted microstructural densification observed in SEM. The most notable evolution occurs in the 1280–1000 cm^−1^ region. The previously broad and complex peaks begin to sharpen and resolve into more distinct and regular profiles. This transformation indicates that the silicoaluminate components are being progressively incorporated into more structurally ordered phases. The formation of better-crystallized C-S-H gel, the increased presence of C-A-H, and the growth of AFt all contribute to a more defined and organized chemical environment for the Si-O-Si and Si-O-Al bonds. Concurrently, the absorption peak near 670 cm^−1^, corresponding to SO_4_^2−^ in AFt, exhibits a marked increase in intensity. This provides clear spectroscopic evidence for the continued and enhanced formation of AFt crystals as the hydration reaction progresses, consuming more aluminate and sulfate phases from the slag and cement [30].

By 28 days, the FTIR spectrum stabilizes, indicating that the major hydration reactions are approaching completion and the chemical structure is maturing. The O-H stretching peak near 3450 cm^−1^ stabilizes in both its width and intensity. This suggests an equilibrium has been reached regarding the formation of hydration products and the associated O-H bonds, as well as the distribution and state of water within the densified matrix. The H-O-H bending vibration peak near 1640 cm^−1^ further weakens and stabilizes at a low intensity. This is characteristic of a mature cementitious system where the microstructure has become highly dense, leaving minimal space for freely vibrating water molecules, most of the water being chemically bound or strongly adsorbed within the fine pores of the C-S-H gel. The peaks in the silicoaluminate region (1280–1000 cm^−1^) are now well-defined, sharp, and exhibit stable intensities. This signifies that the transformation of raw material components into stable, crystalline hydration products is largely complete. The Si-O-Si and Si-O-Al bonds within the C-S-H, C-A-H, and other aluminosilicate phases have established a relatively fixed and ordered configuration, contributing to the mechanical strength and durability of the composite. The SO_4_^2−^ peak near 670 cm^−1^ also stabilizes in intensity, confirming that the formation of AFt has essentially reached its maximum under the given conditions, and its crystalline structure within the matrix is stable. In summary, the FTIR analysis meticulously tracks the chemical evolution from the initial dissolution and amorphous gel formation, through the rapid growth and crystallization of key hydration products, to the final establishment of a stable and chemically mature microstructure. The synergistic participation of oil shale semi-coke and slag is reflected in the progressive changes in the silicoaluminate bonding, which underpins the enhanced mechanical properties observed at the macroscopic scale [31].

#### 3.2.3. Microstructural Analysis of Composite Cementitious Materials

**Figure 8:**
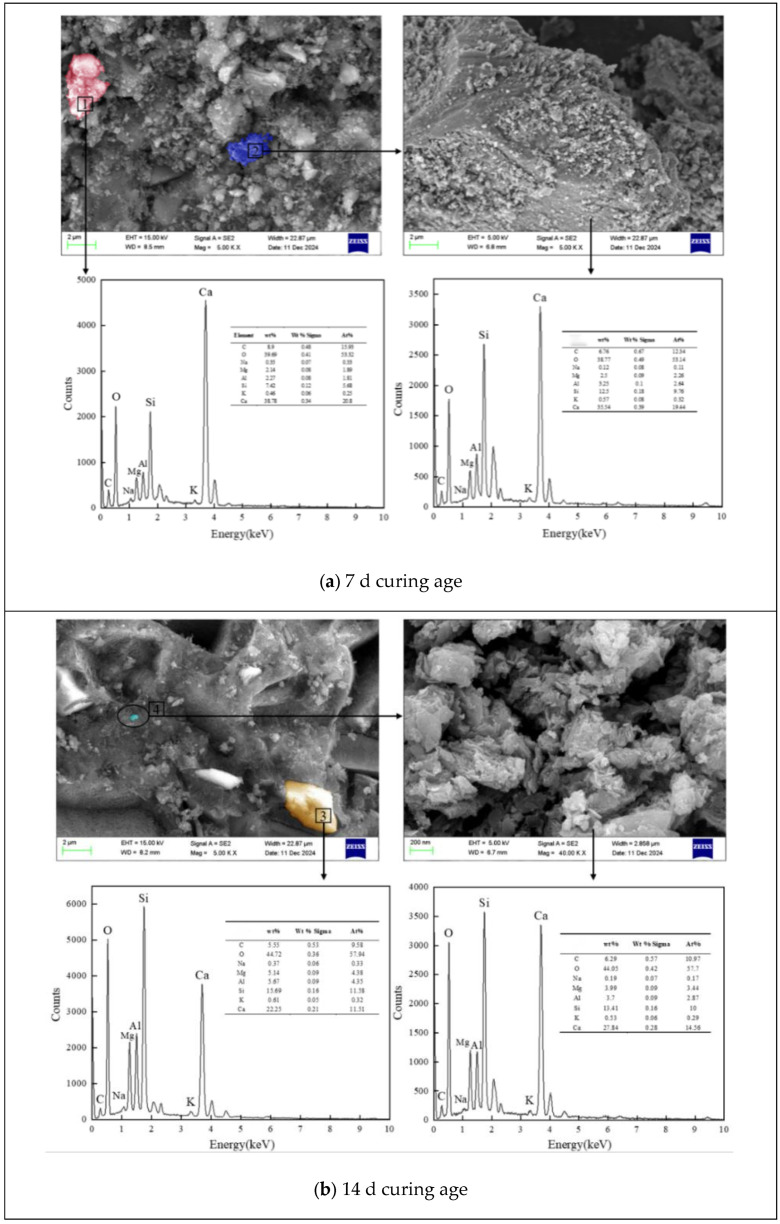

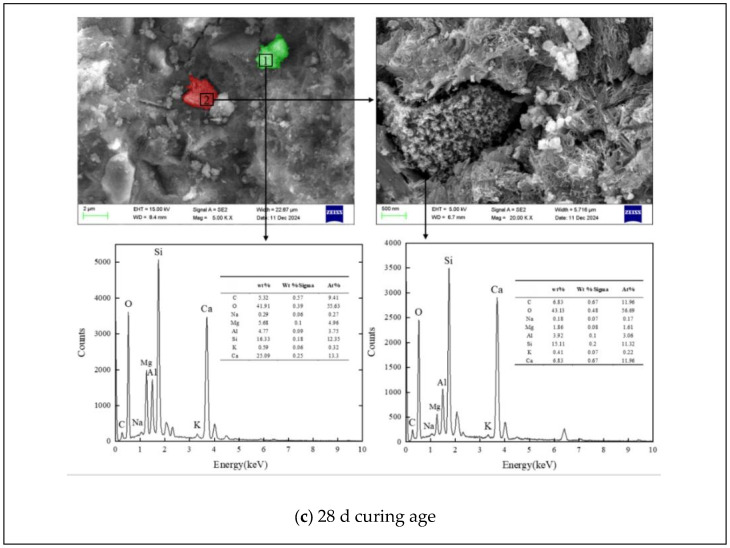
SEM-EDS analysis of composite cementitious materials.

The microstructural evolution and elemental distribution of the oil shale semi-coke-slag composite cementitious material were directly characterized using Scanning Electron Microscopy coupled with Energy-Dispersive X-ray Spectroscopy (SEM-EDS). Specimens cured for 7, 14, and 28 days were examined to correlate morphological and compositional changes with the development of mechanical properties. The results are comprehensively detailed in Figure 8.

The SEM micrograph of the 7-day sample (Figure 8a) reveals a microstructure in its initial stages of formation. The surface morphology appears notably rough and porous, with a network of interconnected pores of varying sizes and shapes. These pores provide the necessary pathways for moisture transport and accommodate the ongoing hydration reactions. At higher magnification, a limited number of hydration products can be discerned. Isolated, short needle-like or rod-shaped crystals, which are characteristic of early-stage ettringite (AFt), are observed sporadically growing on the surfaces of the solid particles. Additionally, some scattered, platy crystals of Portlandite (Ca(OH)_2_) are visible, a direct product of the early hydrolysis of cement clinker minerals. Crucially, a significant portion of the oil shale semi-coke (OSSC) and granulated blast furnace slag (GGBS) particles remain visibly intact, with only their surfaces showing signs of initial dissolution and reaction. The accompanying EDS analysis at this stage detects the major elements Ca, Si, Al, and O. The elemental mapping shows a relatively dispersed distribution of calcium (Ca), while silicon (Si) and aluminum (Al) are predominantly concentrated within the unreacted or partially reacted OSSC and GGBS particles. This microstructural state, dominated by unhydrated particles and high porosity, is directly responsible for the lower mechanical strength measured at 7 days [32].

A remarkable transformation in the microstructure is observed in the 14-day sample (Figure 8b). The overall porosity is significantly reduced compared to the 7-day specimen. Many of the larger pores have been partially filled, and the surface appears noticeably smoother as particles begin to bond more effectively. The most striking development is the emergence of a continuous, interlocking network of hydration products. The quantity of needle-like AFt crystals has increased substantially, and they now appear longer and more interwoven. Concurrently, the formation of platy Ca(OH)_2_ crystals becomes more widespread. These two crystalline phases, along with the amorphous C-S-H gel that acts as a binding matrix, create a cohesive framework that bridges the gaps between solid particles. In this framework, the finer particles of OSSC act as effective micro-fillers, occupying spaces between larger hydration products and slag particles, thereby contributing to the local densification of the matrix. EDS analysis reflects this chemical evolution. The spatial distribution of Ca becomes less uniform and shows signs of enrichment in regions of active hydration product formation. Simultaneously, the relative atomic percentages of Si and Al detected by EDS show a measurable increase, providing direct evidence that the silicoaluminate components from both OSSC and GGBS are being progressively released and incorporated into the growing hydration products, primarily the C-S-H and C-A-H gels.

The microstructure of the 28-day sample (Figure 8c) exhibits a dense and highly integrated morphology. The surface is predominantly compact and block-like, with no obvious large pores, indicating that the hydration products have effectively filled the available space. The particles of cement, OSSC, and GGBS are tightly bound together, creating a virtually monolithic structure. At high magnification, the hydration products are observed to be well-developed and intricately interlocked. The needle-like crystals of C-S-H gel and AFt have grown into elongated, fibrous forms that organize into a robust, three-dimensional skeletal structure. This skeleton provides the primary load-bearing framework of the material. The Ca(OH)_2_ crystals, now fewer in number but larger and thicker, are embedded within this framework. The remaining pores and voids within this crystalline network are densely filled with amorphous hydration gels (C-S-H and C-A-H), which act as a final filler and binder, achieving a high degree of microstructural refinement. The EDS analysis at 28 days confirms the maturation of the system. The elemental distribution of Ca, Si, and Al appears more homogeneous at the micro-scale compared to earlier ages, indicating their widespread incorporation into the hydration products. The continued elevation of Si and Al contents confirms the sustained pozzolanic activity of OSSC and GGBS, consuming Portlandite to form additional cementitious gels. This synergistic reaction between OSSC and GGBS under alkaline activation is the fundamental micro-mechanism responsible for the formation of the dense, stable, and strong microstructure that underlies the excellent mechanical properties of the composite material [33].

#### 3.2.4. Analysis of the Hydration Heat Effect in Composite Cementitious Materials

**Figure 9:**
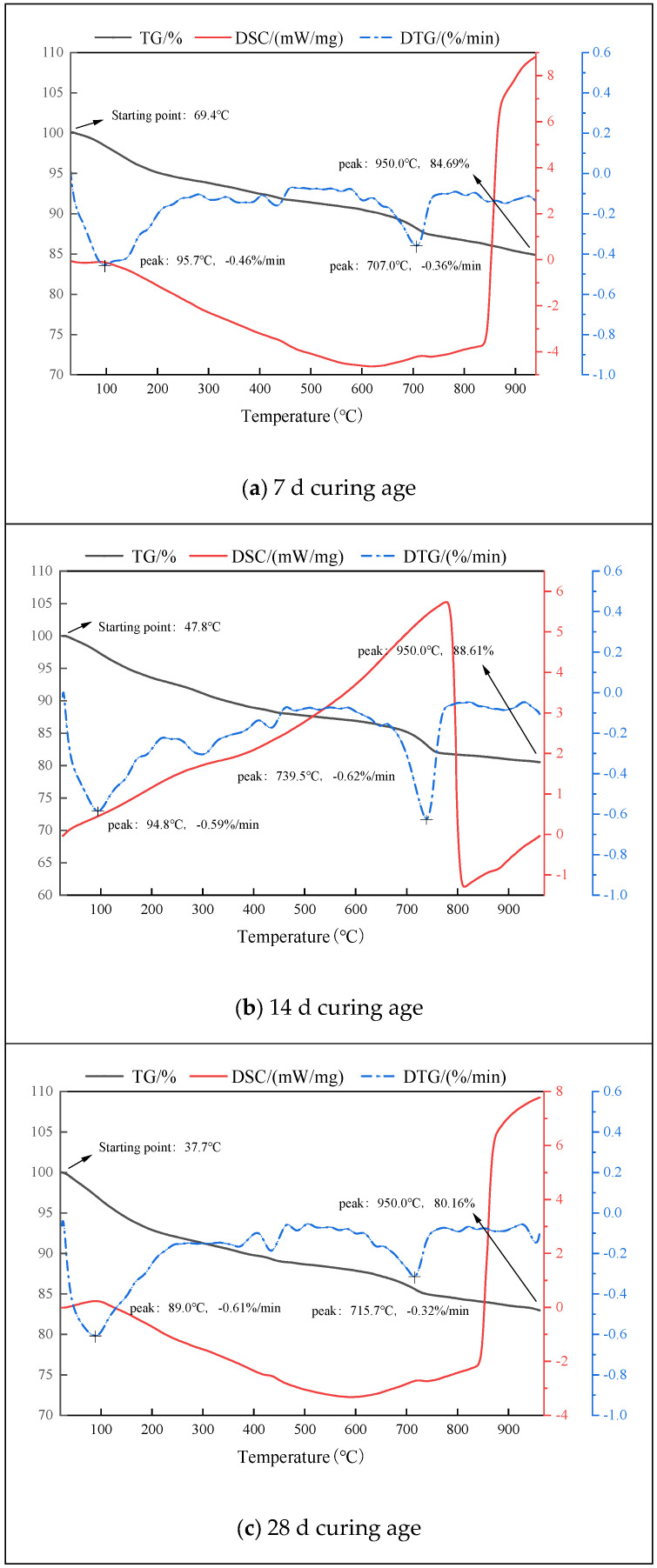
TG-DTG-DSC Curves of Composite Cementitious Materials.

To thoroughly investigate the thermodynamic behavior, mass changes, and reaction kinetics during the hydration process of the oil shale semi-coke-slag composite cementitious material, coupled Thermogravimetric Analysis, Derivative Thermogravimetry, and Differential Scanning Calorimetry (TG-DTG-DSC) was employed. This powerful combination of techniques allows for a comprehensive analysis of key parameters such as onset reaction temperatures, characteristic reaction peaks, and reaction enthalpies at each stage of hydration, thereby revealing the fundamental thermal effects and their influence on the material’s property development. The TG-DTG-DSC curves for samples cured at 7, 14, and 28 days are presented in Figure 9, offering a detailed account of the thermal evolution.

The thermal analysis of the 7-day hydrated sample, as depicted in Figure 9a, captured the system during the initial phase of chemical transformation. The Thermogravimetry (TG) curve commenced mass loss near 69.4 °C, undergoing a gradual yet continuous reduction that culminated in a residual mass of 84.69% at 950 °C. This mass loss profile was not monolithic but could be deconvoluted into several distinct stages, each corresponding to the decomposition of specific components within the composite matrix [34].

Following this initial characterization, the low-temperature stage (approximately 69.4–200 °C) was examined in detail. The mass loss here was primarily attributed to the evaporation of physically adsorbed free water from capillary pores and the removal of loosely bound interlayer water molecules within the poorly crystalline C-S-H gel. Importantly, the relatively modest mass loss in this region indicated that although hydration had commenced, the network of fine pores capable of retaining substantial adsorbed water was not yet fully developed.

Simultaneously, the Derivative Thermogravimetry (DTG) curve provided crucial insights into mass change kinetics. A sharp peak at 95.7 °C, with a mass change rate of −0.46%/min, corresponded to the rapid evolution of adsorbed water. Subsequently, as temperatures escalated into the medium range (~200–500 °C), a prominent broad peak emerged at 707.0 °C (−0.36%/min), which was unequivocally associated with the dehydroxylation of Portlandite (Ca(OH)_2_) [35].

Furthermore, the Differential Scanning Calorimetry (DSC) curve complemented these findings by revealing thermal transitions. It showed a pronounced endothermic peak at low temperatures corresponding to water evaporation and a broader endothermic effect in the medium-high range driven by Ca(OH)_2_ dehydroxylation. The relatively high decomposition temperature suggested the formed Ca(OH)_2_ already possessed considerable crystallinity and structural stability at this early stage.

Building upon these initial observations, the thermal profile of the 14-day sample (Figure 9b) revealed significant evolution. The TG curve now initiated mass loss at a lower onset temperature of 37.7 °C, indicating substantial microstructural refinement. Concurrently, the increased residual mass of 88.16% at 950 °C demonstrated the formation of more stable, non-volatile solid phases that effectively filled the developing pore structure.

Moreover, the DTG curve showed noteworthy changes: a sharper adsorbed water evaporation peak at 89.0 °C (−0.61%/min) suggested altered pore-water interactions, while the Ca(OH)_2_ decomposition peak shifted to 715.7 °C with a decreased mass loss rate (−0.32%/min). These changes collectively indicated both improved Ca(OH)_2_ crystallinity and the onset of its consumption by pozzolanic reactions involving OSSC and GGBS.

Transitioning to the mature system, the 28-day sample (Figure 9c) exhibited more complex behavior. The TG curve began mass loss at an intermediate temperature of 47.8 °C, suggesting pore structure stabilization. However, contrary to expectations, the residual mass decreased to 80.61% at 950 °C, revealing intricate late-stage chemistry where new phase formation occurred alongside the transformation of existing hydrates.

Ultimately, the most revealing changes appeared in the decomposition characteristics. The Ca(OH)_2_ decomposition peak shifted significantly to 739.5 °C with an increased mass loss rate of −0.62%/min, indicating exceptional crystallinity of the remaining crystals. The DSC endothermic peaks consistently aligned with these trends, confirming the continuous evolution of the material’s energy balance. In conclusion, this comprehensive thermal analysis documented a dynamic system where crystalline perfection, pozzolanic consumption, and phase transformations collectively drove microstructural densification.

#### 3.2.5. pH Changes in Composite Cementitious Materials at Different Hydration Ages

**Figure 10:**
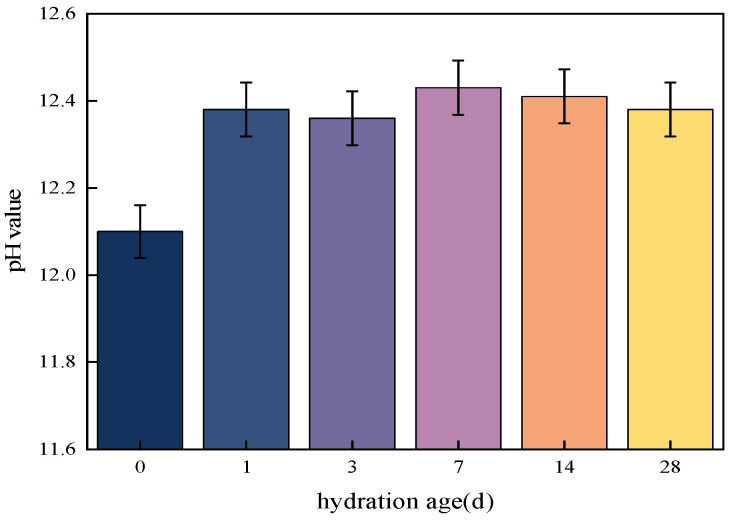
pH Changes in Composite Cementitious Materials at Different Ages.

The evolution of the pore solution pH in cementitious materials is a critical indicator of the chemical environment, directly influencing the dissolution kinetics of solid precursors, the stability of hydration products, and long-term durability. To reveal the dynamic changes in the internal chemical environment of the oil shale semi-coke-slag composite cementitious material and to delve into the physicochemical mechanisms underlying pH variations at different ages, pH measurements were conducted at a series of critical ages: 0 days, 1 day, 3 days, 7 days, 14 days, and 28 days. These ages comprehensively encompass the initial dissolution, rapid development, and relative stabilization phases of the hydration reaction. The pH test results are shown in Figure 10.

The initial pH of the fresh mixture was measured at 12.1, which is inherently alkaline due to the presence of the alkaline activator (water glass). This value underwent a rapid and significant increase, reaching 12.38 after just 1 day of hydration. This sharp rise is predominantly driven by the rapid hydration of the cement clinker minerals, particularly tricalcium silicate (C_3_S). The hydrolysis of C_3_S releases a substantial amount of calcium ions (Ca^2+^) and hydroxide ions (OH^−^) into the pore solution, leading to a swift increase in the OH^−^ concentration and a corresponding jump in pH. This highly alkaline environment (pH > 12.3) is crucial as it is the primary driver for activating the latent hydraulic properties of the granulated blast furnace slag (GGBS) and for initiating the dissolution of reactive silica and alumina from both the slag and the oil shale semi-coke (OSSC). At 3 days, the pH experienced a very slight decrease to 12.36. This minor fluctuation can be attributed to the initial consumption of OH^−^ ions by the early-stage dissolution and reaction of the aluminosilicate components from OSSC and GGBS, as well as potential minor carbonation from atmospheric CO_2_. Despite this slight dip, the pH remained at a very high level, confirming the sustained dominance of cement hydration in controlling the solution chemistry during this initial period.

From 7 days to 14 days, the pH value entered a period of remarkable stability, with readings of 12.43 and 12.41, respectively. The variation between these two time points was minimal. This plateau signifies a dynamic equilibrium established within the system. On one hand, the hydration of cement continues, albeit at a gradually decelerating rate, still contributing to the release of OH^−^ ions and the maintenance of high pH. On the other hand, the dissolution and reaction of OSSC and GGBS are now fully underway. These reactions consume OH^−^ ions: the dissolution of aluminosilicates is favored in a high-pH environment, and the subsequent pozzolanic reactions between these dissolved species and Ca^2+^ (often from the portlandite) further consume hydroxide ions. The near-constant pH during this phase indicates that the rate of OH^−^ generation from ongoing cement hydration is approximately balanced by the rate of OH^−^ consumption by the pozzolanic and hydration reactions of the industrial solid wastes. This stable, highly alkaline environment is optimal for the continued activation and reaction of OSSC and GGBS, facilitating the formation of additional C-S-H, C-A-H, and AFt, as confirmed by the microstructural analyses.

From 14 days to the final measurement at 28 days, a clear, albeit gradual, decreasing trend in pH was observed, with the value dropping from 12.41 to 12.38. This late-stage decline is highly significant and provides key chemical evidence for the long-term synergistic mechanisms. By this stage, the cement hydration reaction has significantly slowed, leading to a reduced rate of new OH^−^ ion production. However, the reactive components in the GGBS and OSSC continue their slow but persistent pozzolanic reactions. These reactions systematically consume the portlandite (Ca(OH)_2_) that was formed during early cement hydration, a process that inherently reduces the OH^−^ concentration in the pore solution. The gradual densification of the microstructure, as seen in SEM, also affects ion mobility, potentially localizing reactions and contributing to the subtle shift in the bulk pH measurement. This slow decrease in pH is therefore a direct chemical signature of the ongoing consumption of alkalis by the prolonged pozzolanic activity, underscoring the sustained synergistic interaction between the cement, slag, and semi-coke that contributes to the long-term microstructural refinement and strength development.

### 3.3. Mechanical Properties of Composite Cementitious Materials

#### 3.3.1. Unconfined Compressive Strength

**Figure 11:**
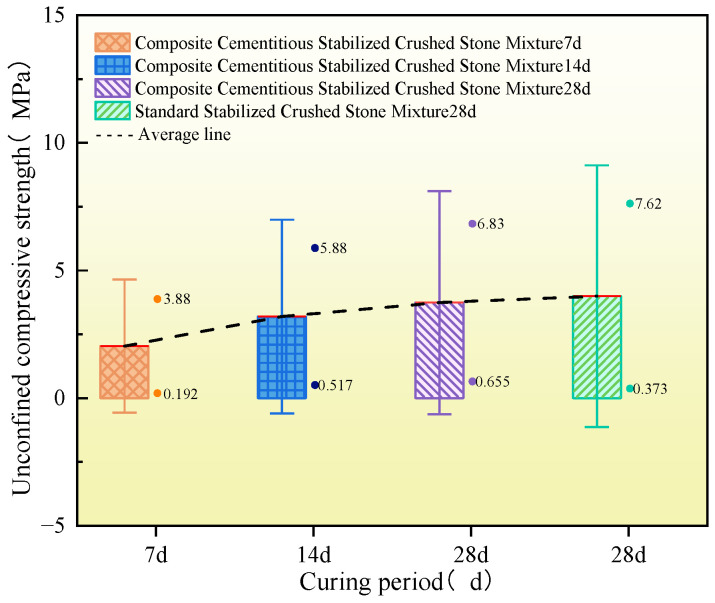
Unconfined compressive strength of stabilized crushed stone mixture using oil shale semi-coke-slag composite cementitious material.

Figure 11 illustrates the development of unconfined compressive strength (UCS) for the stabilized crushed stone mixtures prepared with the optimal OSSC-GGBS formulation. The UCS exhibited a continuous increase with curing age, reaching 3.88 MPa at 7 days, 5.88 MPa at 14 days (a 51.5% increase), and 6.83 MPa at 28 days (a 76.0% increase over 7 days). This strength development is attributed to the ongoing hydration and pozzolanic reactions, which led to the gradual accumulation and densification of hydration products. The 28-day UCS of the OSSC-GGBS mixture reached 89.6% of that of the ordinary cement-stabilized mixture, demonstrating its stable strength performance and significant application potential.

The observed strength development can be explained as follows: Initially, the lower reactivity of OSSC and GGBS compared to cement results in a slower early-age strength gain. The primary clinker phases in cement (C_3_S and C_2_S) hydrate at a controlled rate, with C_2_S contributing more significantly to later-age strength. Concurrently, under alkaline activation, the aluminosilicate components in OSSC and GGBS dissolve, participating in the formation of cementitious products like C-S-H and C-A-H gels. As the curing age increases, these reactions proceed, generating more hydration products that fill the pores and enhance the bonding between the cementitious matrix and aggregates, thereby forming a denser microstructure and increasing the UCS. The rate of strength gain gradually diminishes as the reactions approach completion.

#### 3.3.2. Indirect Tensile Strength

**Figure 12:**
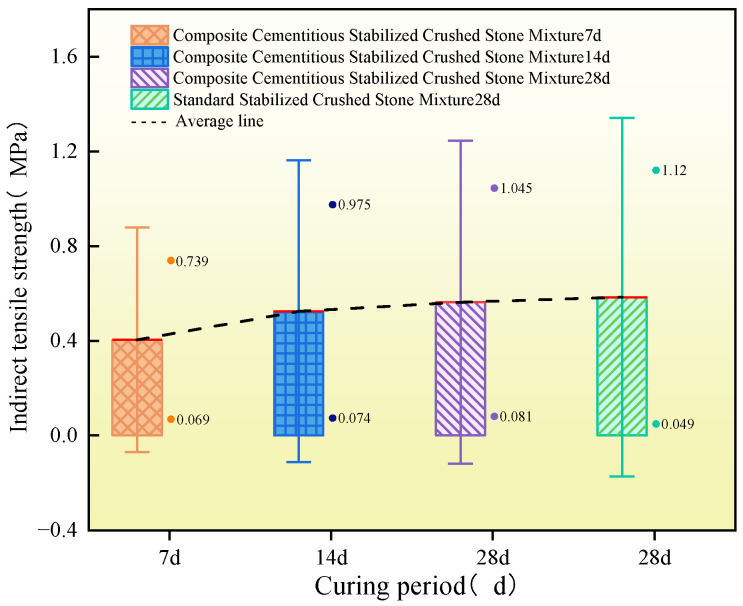
Indirect tensile strength of stabilized crushed stone mixtures using oil shale semi-coke-slag composite cementitious materials.

The indirect tensile strength (ITS), which directly reflects the material’s resistance to cracking, is a critical mechanical indicator for road base applications. The development of ITS for the OSSC-GGBS stabilized mixture is presented in Figure 12.

The ITS exhibited a significant increase with curing age, reaching 0.739 MPa at 28 days, 0.975 MPa at 60 days (a 31.9% increase), and 1.045 MPa at 90 days (a 41.4% increase over 28 days). The low coefficient of variation indicated good consistency in the material’s performance. Notably, the ITS of the composite material consistently reached over 93.3% of that of ordinary cement-stabilized crushed stone, demonstrating high reliability and application potential.

The development of ITS is intrinsically linked to the formation of a cohesive microstructure. The alkali-activated system facilitated the creation of a three-dimensional network of aluminosilicate hydration products. This network, predominantly composed of interwoven C-S-H and C-A-H gels, provides strong adhesion between the cementitious matrix and aggregates. The enhanced bonding and the resulting dense microstructure are key to the material’s improved resistance to tensile stresses and cracking under load.

## 4. Conclusions

(1) Multi-objective optimization was applied to screen the optimal blending proportion of oil shale semi-coke and slag. The composite material attains 94% of the 28 d compressive strength of ordinary Portland cement mortar while reducing material costs by 63.2%. It should be noted that all microstructural and mechanical tests were conducted within a limited curing age range, and the long-term aging evolution law of the material remains to be systematically investigated.
(2) The unconfined compressive strength and splitting tensile strength exhibit excellent statistical consistency, demonstrating that the developed OSSC–GGBS stabilized crushed stone mixture possesses reliable mechanical performance and favorable technological repeatability. However, this study mainly focuses on static mechanical indicators, while key pavement service performances, including dynamic fatigue resistance and frost resistance, still require further experimental verification for practical base application.
(3) Multi-scale characterizations including XRD, FTIR, SEM-EDS and TG-DTG-DSC verify that the strength gain of the composite mainly originates from the rapid dissolution of amorphous aluminosilicate networks in oil shale semi-coke activated by calcium-rich ions released from granulated blast furnace slag. The synergistic reaction between the two solid wastes accelerates the formation of cross-linked C-(A)-S-H gels, constructing a dense matrix and greatly enhancing interfacial bonding strength. In follow-up research, statistical indicators such as standard deviation and coefficient of variation will be adopted to improve data analysis, combined with molecular dynamics simulations to quantify the crystallization kinetics of mineral phases and the data dispersion of multi-batch specimens.

## Figures and Tables

**Table 1:** Chemical compositions of raw materials.

Component	Unit	SiO_2_	CaO	Al_2_O_3_	MgO	SO_3_	TiO_2_	BaO	Fe_2_O_3_	K_2_O	Na_2_O
Semi-coke	(%)	53.62	3.60	29.29	2.30	0.66	1.12	0	0	0	0
Slag	(%)	37.21	34.21	12.32	8.51	2.53	1.19	1.15	0	0	0
Cement	(%)	23.91	57.66	6.64	2.44	4.07	0	0	3.17	0.93	0.67

**Table 2:** Quality inspection of water glass.

Inspection Items	Unit	Standard Requirements	Test Results
Appearance	-	Colorless or slightly tinted transparent/translucent viscous liquid	Light-colored, transparent, viscous liquid
Na_2_O Content	%	≥12.8	13.73
SiO_2_ Content	%	≥29.2	32.35
Modulus	-	2.20–2.50	2.43
Baume degree (20 °C)	°Bé	48.5–52.5	50

**Table 3:** Mix proportions of the composite cementitious materials.

Group	Cement Content (%)	OSSC:GGBS Mass Ratio	Water Glass Modulus	Water Glass Dosage (%)	Water-to-Binder Ratio
1	15	4:6	1.4	10	0.42
2	15	3:7	1.4	10	0.42
3	15	2:8	1.4	10	0.42

**Table 4:** Criteria for Optimizing Response.

Factor/Response	Goal	1		2		Importance (+++ Represents Importance)
		Lower	Upper	Lower	Upper	
A: (OSSC:GGBS Ratio)	Range	6:4	8:2	6:4	8:2	+++
B: Cement Content (%)	Range	10%	20%	10%	20%	+++
C: Water Glass Modulus	Range	1.3	1.5	1.3	1.5	+++
D: Water Glass Dosage (%)	Range	8%	12%	8%	12%	+++
Y_1_: Compressive Strength (MPa)	Maximize	26.6	37	26.6	37	+++
Y_2_: Flexural Strength (MPa)	Maximize	4.5	6.6	4.5	6.6	+++

**Table 5:** Experimental validation results for the optimized mix designs.

Response Combination	Predicted Value	Measured Value	Error (%)
Combination 1 (OSSC:GGBS = 8:2, Cement = 20%, Modulus = 1.4, Dosage = 10%)	-	-	-
Compressive Strength (MPa)	37.00	37.10	0.27
Flexural Strength (MPa)	7.20	7.22	0.27
Combination 2 (OSSC:GGBS = 3:7, Cement = 15%, Modulus = 1.4, Dosage = 10%)	-	-	-
Compressive Strength (MPa)	37.37	37.50	0.35
Flexural Strength (MPa)	7.05	7.04	0.14

**Table 6:** Properties and costs of different cementitious materials.

Cementitious Material	Cost (RMB/t)	7-Day Strength (MPa)	28-Day Strength (MPa)
Ordinary Portland Cement (PO 42.5)	450	23.52	39.3
OSSC-GGBS Combination 1	188.8	20.80	37.1
OSSC-GGBS Combination 2 (Optimal)	165.4	21.0	37.5

## Data Availability

The original contributions presented in this study are included in the article. Further inquiries can be directed to the corresponding authors.
